# Multi-Fractal Weibull Adaptive Model for the Remaining Useful Life Prediction of Electric Vehicle Lithium Batteries

**DOI:** 10.3390/e25040646

**Published:** 2023-04-12

**Authors:** Wujin Deng, Yan Gao, Jianxue Chen, Aleksey Kudreyko, Carlo Cattani, Enrico Zio, Wanqing Song

**Affiliations:** 1 School of Electronic & Electrical Engineering, Shanghai University of Engineering Science, Shanghai 201620, China; dwj058@sina.com (W.D.); gy@sues.edu.cn (Y.G.); merlyn21@126.com (J.C.); swqls@126.com (W.S.); 2 Department of Medical Physics and Informatics, Bashkir State Medical University, Lenina St. 3, 450008 Ufa, Russia; akudreyko@bashgmu.ru; 3 Engineering School, DEIM, University of Tuscia, 01100 Viterbo, Italy; 4 The Centre for Research on Risk and Crises (CRC) of Ecole de Mines, Paris Sciences & Lettres (PSL) University, 06904 Paris, France; enrico.zio@polimi.it; 5 Energy Department, Politecnico di Milano, Via La Masa 34/3, 20156 Milan, Italy

**Keywords:** electric vehicle lithium battery, remaining useful life, multi-fractal Weibull motion, long-range dependence, 1/f noise, age and state-dependent adaptive model

## Abstract

In this paper, an adaptive remaining useful life prediction model is proposed for electric vehicle lithium batteries. Capacity degradation of the electric car lithium batteries is modeled by the multi-fractal Weibull motion. The varying degree of long-range dependence and the 1/f characteristics in the frequency domain are also analyzed. The age and state-dependent degradation model is derived, with the associated adaptive drift and diffusion coefficients. The adaptive mechanism considers the quantitative relations between the drift and diffusion coefficients. The unit-to-unit variability is considered a random variable. To facilitate the application, the convergence of the RUL prediction model is proved. Replacement of the lithium battery in the electric car is recommended according to the remaining useful life prediction results. The effectiveness of the proposed model is shown in the case study.

## 1. Introduction

### 1.1. Research Background

**Figure 1:**
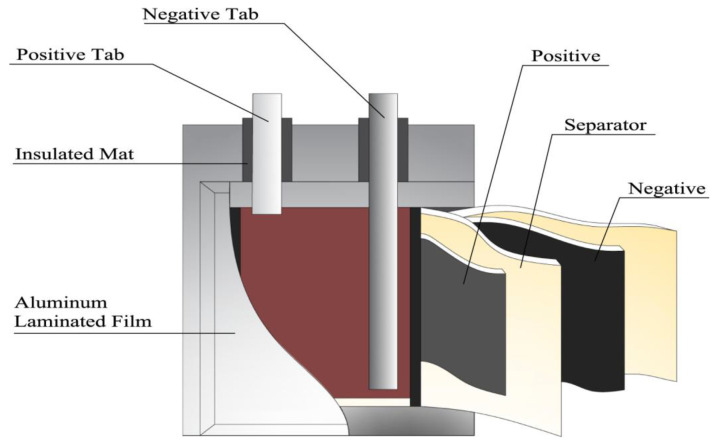
The schematic structure of the lithium battery.

Recently, electric cars powered by lithium batteries are attracting more and more interest from consumers [1]. The schematic structure of the lithium batteries used in the electric vehicle is in Figure 1 [2]. The main components of lithium batteries are the negative electrode, positive electrode, separator and packeting. The electric energy and the chemical energy convert with each other in the charging and discharging process.

**Figure 2:**
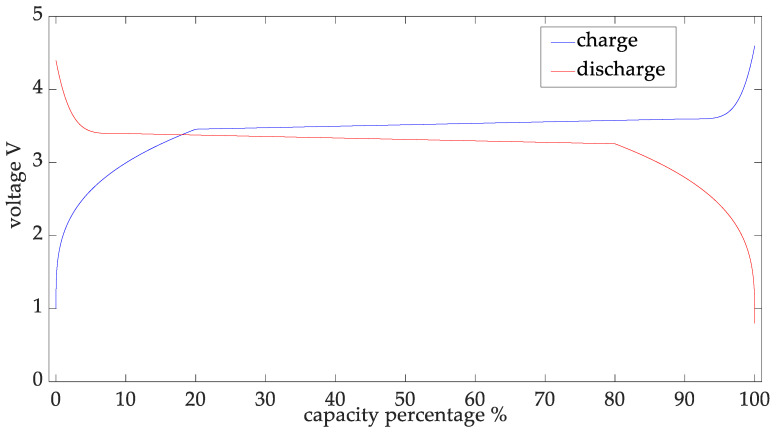
Charging and discharging characteristics in a typical lithium battery cycle.

In the charging stage of the battery cycle, the electric car is plugged into the power system to absorb electricity as a kind of single-phase load [3]. In the discharging stage, the electricity is discharged from the battery to power the electric motor [4]. A typical relationship between the lithium battery capacity and the voltage is presented in Figure 2 with respect to a single cycle of charge–discharge [5]. At the beginning and at the end, the voltage fluctuates heavily for both the charging and discharging stages. When the voltage is in the steady phase, the electric car is functioning in the desired way.

### 1.2. Significance of the Research

**Figure 3:**
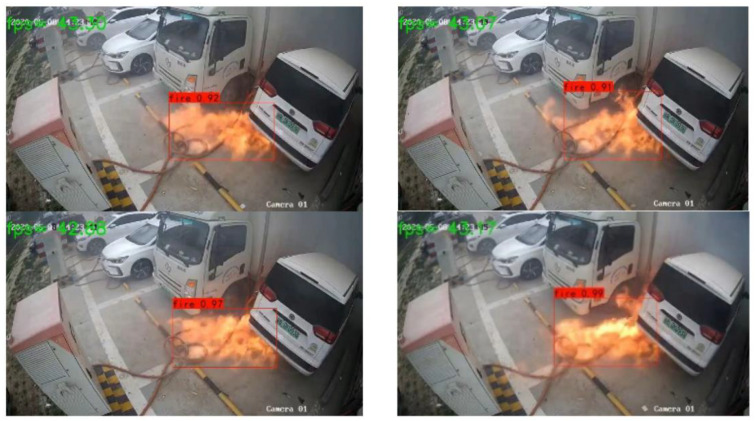
Fire accident in an electric vehicle charging station.

The charging of electric cars exposes people to risks and can impact the reliability of the power system. Figure 3 presents a series of screenshots of an electric vehicle charging station [6]. The charging accident may cause the burndown of the whole station. 

The main cause of dangerous accidents during the electric car charging stage is the aging and failure of the lithium battery [7]. The degradation of the lithium battery can cause thermal runaway, short circuits, and even an explosion of the plugged-in electric car. The thermal and mechanical stresses during the charging stage result in degradation and damage to the materials of the anode and cathode. The growth of Li dendrite in the anode during the charging process can compromise the insulation of the battery. The sharp metallic lithium can penetrate the insulation and cause a devastating short circuit. 

An effective way to control such risk is preventive battery replacement. The over-aged and risk-exposed lithium batteries need to be replaced before the accident occurs. To this aim, data collected from the embedded sensors are used to predict the probability density function (pdf) of the remaining useful life (RUL) [8,9]. Based on the RUL prediction results of the lithium battery, the driver of the electric car can be recommended to replace the battery [10]. It is commonly accepted that the battery should be replaced when the impedance doubles and the capacity decreases to 80% of the rated value [11]. Therefore, we can consider the capacity and the impedance as the health indicator (HI) for the lithium battery in the RUL prediction process. 

### 1.3. Literature Review

Methods for RUL prediction of Lithium batteries can be divided into two main categories: model-based and data-driven approaches. The model-based approaches are based on the physical and electrochemical theory of the lithium battery. The simulation of the degradation evolution may not be accurate because of the complexity and nonlinearity of the process. To overcome this, the error correction procedure is applied in [12]. The data-driven approaches extract the degradation information from historical aging data and predict the future degradation evolution with artificial intelligence algorithms and stochastic processes. It should be noted that the data-driven RUL prediction model follows the assumption that the training data and the test data are similar in distribution [13]. 

Support vector regression and classification are combined in an RUL prediction algorithm for the lithium battery: the classification model provides a gross estimation of the degradation trend, and the regression model is then used to accurately predict the RUL when the battery is close to the end of life (EOL) [14]. Fuzzy information granulation and linguistic description are applied to address some limitations of RUL prediction methods for lithium batteries [15]. Ref [16] proposes a flexible and effective online training strategy by relevance vector machine for incremental optimization to enhance prediction ability. An RUL prediction method for lithium batteries is proposed based on Gaussian mixture regression and an auto-encoder [17]. A novel multi-hierarchy network based on multi-ordered neurons, namely, cocktail long short-term memory network (C-LSTM), is proposed for the RUL prediction of the mechanical parts [18]. In [19], data of high quality are generated by convolutional recurrent generative adversary networks to compensate for the shortage of data. Considering the parallel integration of the spatial and temporal features, ref [20] proposes a parallel hybrid neural network for the RUL prediction based on multi-sensor data. In engineering application, acquiring sufficient data may be difficult. For the cross-domain condition of the RUL prediction, a variational local weighted deep sub-domain adaptation network is proposed [21]. 

RUL prediction models based on the stochastic processes are formulated as the sum of a drift term and a diffusion term [22]. The drift term contains a drift function and a drift coefficient. The drift function is usually nonlinear and describes the degradation rate. The drift coefficient describes the dispersion of the drift function. The diffusion term is driven by the stochastic process to characterize the temporal variability. The stochastic processes have different assumptions on the data distributions. The Weibull distribution, Gamma distribution, log-normal distribution and exponential distribution are often used in RUL prediction [23]. In this paper, we take the Weibull distribution to model the data distribution for its better-fitting results.

The Brownian motion is suitable to describe the non-monotonous degradation process [24]. However, the Brownian motion is Markovian, whereas the degradation data often show non-Markovian characteristics, e.g., the capacity data of the lithium battery possesses long-range dependence [25]. Long-range dependence means that the present value is influenced by the previous values of the time series. The process and time series are long-range dependent if the Hurst exponent takes value in (0.5, 1). The stochastic process with long-range dependence is also a 1/f noise in the frequency domain [26]. Combined with the Karman filter and the expectation maximation algorithm, the strong Markovian characteristics of Brownian motion can be improved [27]. To track the dynamics and multi-source variability of a degradation process together, a general time-varying Wiener process (GTWP) is proposed in [28]. Ref. [29] proposes a nonlinear degradation model based on fractional Brownian motion with dynamic properties (FBM-D) to feature the long-range dependence of the lithium battery degradation data. 

### 1.4. Recent Progress about the RUL Prediction Based on Random Process

The multifractional Brownian motion is derived by changing the constant Hurst exponent in the FBM into a variable function [30]. The linear multifractional Lévy stable motion is utilized to derive a RUL prediction method in [31]. The Hurst exponent in the model varies. The purpose of the generalization is that the fractal properties in the degradation change with varying operation conditions.

Currently, the nonlinear drift functions in the degradation models are mostly age dependent. However, in practical systems, the deteriorating rate changes according to the degradation status [32]. In [33], a degradation model depending on both age and state are proposed, with unit-to-unit variability. The unit-to-unit variability is defined as the change of degradation rates in the different health states, and it is expressed by a random coefficient in the nonlinear degradation model. 

The degradation rate of the lithium battery is constantly changing in the different degradation modes [34]. Therefore, the drift function should be able to change constantly based on historical information. Ref. [35] describes the adaptation of the drift term with the evolution of random walk, which implies that the degradation rate is usually increasing. Evidence shows that the noise variable can adaptively alleviate the difficulty of the data-driven approach caused by the difference in data distributions.

Previous adaptive prediction models only consider the adaptation of the drift coefficient and assume the diffusion coefficient to be constant, which is not realistic. When the degradation is fast, the temporal variation is also strong. Ref. [36] proposes a degradation model in which the diffusion coefficient and drift coefficient are linearly correlated. The experiment results also confirm the linear correlation theory. The proportional relationship is derived based on the acceleration factor constant principle [37]. The acceleration factor is used to calculate the RUL in stressed conditions based on the RUL in normal conditions. Considering their quantitative relationship, both the drift and diffusion coefficients are adaptive with the random walk-in Ref. [38].

### 1.5. Contributions and Structure of the Article

The properties of the multi-fractal Weibull motion (mfWm) are analyzed, e.g., long-range dependence and 1/f characteristics. With varying Hurst exponent, the mfWm can express the varying degree of long-range dependence in the actual degradation. 

An age and state-dependent degradation model is presented in the paper. The random coefficient in the drift function accounts for the unit-to-unit variability. The nonlinearity of the degradation is fitted with the power function. The linear relationship between the drift and diffusion coefficients is considered so that both coefficients can be adaptively updated. The adaptive RUL prediction method is then derived with the convergence proven.

The rest of the paper is arranged as follows. Section 2 provides proof of long-range dependence and 1/f characteristics for the mfWm. In Section 3, the age and state-dependent degradation model is derived. The adaptive mechanism for both the drift and diffusion coefficients is proposed. In the case study, the RUL prediction for the lithium battery is provided. The works of the paper are summarized in the conclusion.

## 2. Long Range Dependence and 1/f Characteristics of the mfWm

### 2.1. Definition of the mfWm

The pdf of the Weibull distribution is:

(1) $$f(x|\lambda ,k)=\frac{k}{\lambda }{\left(\frac{x}{\lambda }\right)}^{k-1}\exp\left\{-{\left(\frac{x}{\lambda }\right)}^{k}\right\},x\geq 0,$$

where λ is the scaling parameter and k is the shape parameter. 

The mfWm is derived with the Riemann-Liouville integral. In the mfWm, the Hurst exponent H is replaced by the random variable $H_{v}$:

(2) $$mfWm(t)=\frac{1}{\Gamma (H_{v}+0.5)}\int_{0}^{t}{\left(t-u\right)}^{H_{v}-0.5}dWp(u),$$

where $H_{v}∼U(0.5,1)$ and Wp is the white Weibull noise.

Defining a function φ(t), the mfWm can be viewed as a convolution operation:

(3) $$\varphi (t)=\frac{t^{H_{v}-0.5}}{\Gamma (H_{v}+0.5)},$$

(4) $$\begin{array}{cc} mfWm(t) & =\int_{0}^{t}\frac{{\left(t-u\right)}^{H_{v}-0.5}}{\Gamma (H_{v}+0.5)}\frac{dWp(u)}{du}du\\ & =\frac{dWp(t)}{dt}*\frac{t^{H_{v}-0.5}}{\Gamma (H_{v}+0.5)},\\ & =\frac{dWp(t)}{dt}*\varphi (t), \end{array}$$

where $H_{v}$ is the Hurst variable and Γ is the Gamma function.

**Figure 4:**
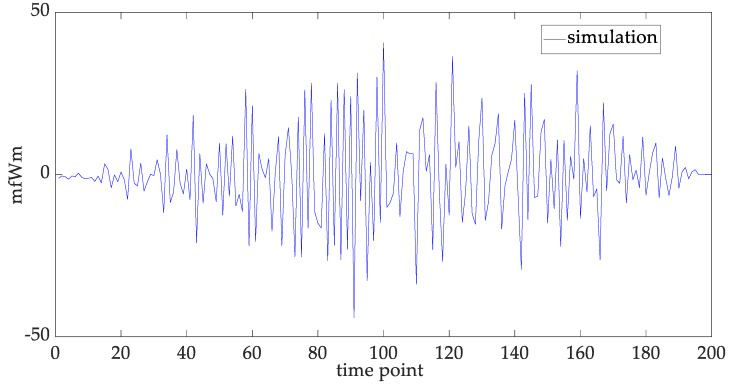
Time series of the mfWm.

Therefore, the mfWm can be considered the output of a linear system. The input signal is the derivative of the white Weibull noise, and the impulse response is φ(t). The exemplary trajectory of the mfWm is plotted in Figure 4. The augmented Dickey–Fuller test proves that the mfWm is a stationary stochastic process [39]. The mean and variance of the mfWm can be calculated, which means that the mfWm is also a wide stationary stochastic process [40]. 

### 2.2. The mfWm Is a Long Range Dependent 1/f Noise

The integration of the autocorrelation function for the long-range dependent time series is divergent:

(5) $$\int_{0}^{\infty }autoC(\tau )d\tau =\infty ,$$

where autoC(τ) is the autocorrelation function and τ is the time delay.

The reason for the divergence is that the autocorrelation function is decaying in power law, which implies that the autocorrelation is strong for long time delays:

(6) $$autoC(\tau )\sim \tau^{-\beta },$$

where β is positive and represents the decaying rate.

The power spectrum density (PSD) is the Fourier transformation of the autocorrelation function for a wide stationary stochastic process according to the Venasinchin theorem [41]:

(7) $$\begin{array}{c} PSD(f)=Fourier(autoC(\tau ))\\ =\int_{0}^{\infty }autoC(\tau )\exp(-2\pi jf\tau )d\tau , \end{array}$$

where f is the frequency and j is the imaginary unit.

The stochastic process is 1/f noise if the PSD follows the power law decay:

(8) $$PSD(f)\propto \frac{1}{f^{\alpha }},$$

where α∈(0,2) determines the decaying rate.

The long-range dependence in the time domain is equivalent to the 1/f characteristics in the frequency domain:

(9) $$\underset{f\to 0}{\lim}PSD(f)=\int_{0}^{\infty }autoC(\tau )d\tau \;\propto \underset{f\to 0}{\lim}\frac{1}{f^{\alpha }}=\infty .$$

In this section, the mfWm is proven to be a long-range dependent 1/f noise.

**Theorem** **1.** *The mfWm is a long-range dependent 1/f noise.*

**Proof of Theorem** **1.** We can rewrite the definition of the mfWm as Equation (10):

(10) $$mfWm(t)=\int_{0}^{\infty }\frac{{\left(t-u\right)}^{H_{v}-0.5}}{\Gamma (H_{v}+0.5)}\frac{dWp(u)}{du}U(t-u)du,$$

where $U(t-u)=\{\begin{array}{c} 1,u<t\\ 0,u>t \end{array}$.

According to the theory of linear systems, the following equations of input X(t) and output Y(t) can be formulated with respect to the impulse response φ(t):

(11) $$crossC(X(t_{1}),Y(t_{2}))=autoC(X(t_{1}),X(t_{2}))*\varphi (t_{2}),$$

(12) $$autoC(Y(t_{1}),Y(t_{2}))=crossC(X(t_{1}),Y(t_{2}))*\varphi (t_{1}),$$

where crossC(τ) is the cross-correlation function.

The autocorrelation function can be calculated with convolution. The autocorrelation function of the white Weibull noise is the Dirac function, which is in Equation (13). Then the autocorrelation for the derivative of the white Weibull noise can be calculated in Equation (14).

(13) autoC(Wp(τ))=Wp(τ)∗Wp(−τ)=δ(τ),

(14) $$autoC(\frac{dWp(\tau )}{d(\tau )})=\frac{dWp(\tau )}{d(\tau )}*\frac{dWp(-\tau )}{d(-\tau )}=\ddot{\delta }(\tau ),$$

where δ(τ) is the Dirac function and $\ddot{\delta }(\tau )$ is the second derivative of the Dirac function.

Therefore, the cross correlation can be calculated:

(15) $$\begin{array}{cc} crossC(X(t_{1}),Y(t_{2})) & =\int_{0}^{\infty }autoC(X(t_{1}),X(s))\varphi (t_{2}-s)ds\\ & =\int_{0}^{\infty }\ddot{\delta }(t_{1}-s)\frac{{\left(t_{2}-s\right)}^{H_{v}-0.5}U(t_{2}-s)}{\Gamma (H_{v}+0.5)}ds\\ & ={\left[\frac{{\left(t_{2}-s\right)}^{H_{v}-0.5}U(t_{2}-s)}{\Gamma (H_{v}+0.5)}\right]}^{\prime \prime }{}_{s=t_{1}}\\ & =\frac{\left(H_{v}-0.5)(H_{v}-1.5)U(t_{2}-t_{1}\right)}{\Gamma (H_{v}+0.5)}{\left(t_{2}-t_{1}\right)}^{H_{v}-2.5}, \end{array}$$

where Γ is the Gamma function, $H_{v}$ is the Hurst variable and U is the step function.

The autocorrelation function of the mfWm is:

(16) $$\begin{array}{c} autoC(Y(t_{1}),Y(t_{2}))=\int_{0}^{\infty }crossC(X(s),Y(t_{2}))\varphi (t_{1}-s)ds\\ =\int_{0}^{\infty }\frac{U(t_{1}-s)U(t_{2}-s)(H_{v}-0.5)(H_{v}-1.5)}{\Gamma^{2}(H_{v}+0.5)}{\left(t_{2}-s\right)}^{H_{v}-2.5}{\left(t_{1}-s\right)}^{H_{v}-0.5}ds \end{array}.$$

Define $t=\min(t_{1},t_{2})$ and then we can discuss the autocorrelation function in the interval of (0,t):

(17) $$\begin{array}{cc} autoC(t) & =\frac{\left(H_{v}-0.5)(H_{v}-1.5\right)}{\Gamma^{2}(H_{v}+0.5)}\int_{0}^{t}{\left(t-s\right)}^{2H_{v}-3}ds\\ & \overset{t-s=x}{\to }\frac{\left(H_{v}-0.5)(H_{v}-1.5\right)}{\Gamma^{2}(H_{v}+0.5)}\int_{0}^{t}x^{2H_{v}-3}dx\\ & =\frac{\left(H_{v}-0.5)(H_{v}-1.5\right)}{\Gamma^{2}(H_{v}+0.5)(2H_{v}-2)}t^{2H_{v}-2}, \end{array}$$

where $2H_{v}-2\in (-1,0)$.

When the time is within (0,t), the time delay τ is the same as the current time. Therefore, we can rewrite Equation (17) as:

(18) $$autoC(\tau )=\frac{\left(H_{v}-0.5)(H_{v}-1.5\right)}{\Gamma^{2}(H_{v}+0.5)(2H_{v}-2)}\tau^{2H_{v}-2}.$$

The autocorrelation of the mfWm is decaying in the power law, and therefore it is long-range dependent. The PSD of mfWm is the Fourier transform of the autocorrelation:

(19) $$\begin{array}{cc} PSD(f) & =\frac{\left(H_{v}-0.5)(H_{v}-1.5\right)}{\Gamma^{2}(H_{v}+0.5)(2H_{v}-2)}\int_{0}^{\infty }\tau^{2H_{v}-2}\exp\{-2\pi jf\tau \}d\tau \\ & \overset{2\pi jf\tau =x}{\to }\frac{(H_{v}-0.5)(H_{v}-1.5){\left(-j\right)}^{2H_{v}-1}}{\Gamma^{2}(H_{v}+0.5)(2H_{v}-2){\left(2\pi f\right)}^{2H_{v}-1}}\int_{0}^{\infty }x^{2H_{v}-2}\exp\{-x\}dx\\ & =\frac{(H_{v}-0.5)(H_{v}-1.5)\Gamma (2H_{v}-3){\left(-j\right)}^{2H_{v}-1}}{\Gamma^{2}(H_{v}+0.5)(2H_{v}-2){\left(2\pi f\right)}^{2H_{v}-1}}\\ & \sim \frac{1}{f^{2H_{v}-1}}, \end{array}$$

where $2H_{v}-1\in (0,1)$.

Thus, we have proven that the mfWm is also a 1/f noise. In conclusion, the mfWm is a long-range dependent 1/f noise. □

## 3. Age and State-Dependent Degradation Model with Adaptive Mechanism

### 3.1. Age and State-Dependent Drift Function

In the degradation model, the drift function is utilized to quantify the deteriorating rate, which is the deterministic characteristic of the degradation. The selection of the drift function is of great importance for the performance of the degradation model [42]. The age-dependent degradation function can be chosen as the linear function, power function and exponential function [43,44,45]. 

With different parameters, the power function can mimic the degradation trend of the linear function and exponential function. Therefore, we choose the power function to describe the temporal nonlinearity of the degradation. 

The state also impacts the degradation; thus, the state should be included in the drift function. The age and state-dependent degradation model proposed by the previous literature [46]:

(20) $$\eta (X(t),t,\theta )=aX(t)+bct^{c-1},$$

where c expresses the degradation speed. a and b are the impact factor of state and age separately. The formulation of Equation (20) indicates that the impact of age and state to the degradation are equally important to the degradation. 

The aging state is constantly changing, which affects the degradation rate. Therefore, the unit-to-unit variability needs to be considered. The parameter $a\sim N(\mu_{a},\sigma_{a}{}^{2})$ is constantly changing during the degradation process to express the unit-to-unit variability. The parameters b and c are fixed, which features the general degradation speed with the nonlinear power function. 

Coefficients should be added to balance the effects of age and state, which sum to be one. The improved age and state-dependent drift function is:

(21) $$\eta (X(t),t,\theta )=0.5(aX(t)+bct^{c-1}),$$

where the balance coefficient 0.5 is chosen because the age and state are equally important to the degradation. 

### 3.2. Adaptive Updates of the Degradation Model 

The degradation model is presented below in Equation (22), and the mfWm serves as the temporal variability in the diffusion term of the degradation model:

(22) $$\begin{array}{cc} X(t) & =X(0)+\mu \int_{0}^{t-1}\eta (X(s),s,\theta )ds+\sigma_{H}mfWm(t)\\ & =X(0)+0.5\mu \int_{0}^{t-1}(aX(s)+bcs^{c-1})ds+\sigma_{H}mfWm(t)\\ & =X(0)+0.5\mu a\int_{0}^{t-1}X(s)ds+0.5\mu b{\left(t-1\right)}^{c}+\sigma_{H}mfWm(t), \end{array}$$

where $\sigma_{H}$ is the diffusion coefficient and μ is the drift coefficient. The initial value of the degradation model is zero.

Take the linear correlation between the drift and diffusion coefficients into consideration:

(23) $$X(t)=X(0)+0.5\mu a\int_{0}^{t-1}X(s)ds+0.5\mu b{\left(t-1\right)}^{c}+\kappa \mu mfWm(t),$$

where $\kappa \sim N(\mu_{\kappa },\sigma_{\kappa }{}^{2})$.

The increment between two adjacent time points is calculated as:

(24) $$\begin{array}{cc} \Delta X(t-1) & =X(t)-X(t-1)\\ & =0.5\mu a\int_{t-2}^{t-1}X(s)ds+0.5\mu b\Delta (t^{c})+\kappa \mu mfWm(\Delta t), \end{array}$$

where $\Delta (t^{c})={\left(t-1\right)}^{c}-{\left(t-2\right)}^{c}$ and mfWm(Δt)=mfWm(t)−mfWm(t−1).

The drift coefficient controls the dispersion of the degradation rate measured with the drift function. Considering the stochasticity of the degradation, the drift coefficient needs to be adaptively updated. The diffusion coefficient can also be updated due to the linear correlation. The adaptive mechanism is:

(25) $$\{\begin{array}{c} \mu (t)=\mu (t-1)+\varepsilon (t),\\ \sigma_{H}(t)=\kappa \mu (t),\\ X(t)=X(t-1)+0.5\mu (t-1)a\int_{t-2}^{t-1}X(s)ds+0.5\mu (t-1)b\Delta (t^{c})+\sigma_{H}(t-1)mfWm(\Delta t), \end{array}t\in 1,2,\ldots$$

where $\varepsilon (t)\sim N(0,\sigma_{\mu }{}^{2})$ and μ(0)=1.

The drift coefficient in Equation (25) is considered to be a time-dependent function. The evolution is based on the random walk, which provides stochasticity to the degradation speed. The purpose is to improve the robustness of the model when the future degradation trend is significantly different from the historical data. The drift coefficient can adjust the degradation model to future changes with dispersion. With the adaptation of the random walk, the robust effect of the degradation model becomes stronger. At the beginning of the evolution, the degradation speed is the same as the drift function derived from historical knowledge. Therefore, the initial value of the drift coefficient is one. The mean of the Gaussian white noise is zero to provide an asymmetric dispersion opportunity for the drift function.

### 3.3. Analysis of the Degradation Model for Lithium Batteries Powering the Electric Motor

The proposed degradation model is adaptive in two aspects. First, the degree of long-range dependence in the degradation model changes frequently due to the multi-fractal characteristics of the mfWm. This is different from previous degradation models considering the fractional stochastic process with the uniform Hurst exponent, which contradicts reality. Indeed, the fractal properties of the degradation process change frequently with the varying operation conditions. The RUL prediction model based on the mfWm is, instead, adaptive to the varying statistical properties of the degradation. Second, the drift and diffusion coefficients are adaptive to the change of modes within the degradation process, which means that the degradation rate and the variational speed vary during the degradation process.

An electric motor converts electrical energy into mechanical energy in the normal rotation and otherwise in the sudden brake. This energy exchange between the battery and electric motor contributes to a stronger recovery effect compared with other kinds of electric load [47]. The recovery effect should be considered in the RUL prediction of lithium batteries for the electric vehicle [48]. The degradation rate and the variational speed in the normal discharging mode and the regenerative braking mode are different. Therefore, adaptive updates for the drift coefficient and diffusion coefficient are proposed. The operation condition for the electric motor in the electric car is quite complex, e.g., the motor stall, compared with other kinds of electrical load [49]. The fractal characteristics of the degradation data are changing constantly due to the varying operation condition. In summary, the impact of age and state should not be ignored for the lithium battery powering the electric motor. Thus, the age and state-dependent drift function is selected for this work.

### 3.4. Parameter Estimation 

The capacity degradation data is decreasing with the initial value to be the maximum. With the data preprocessing technique, the degradation trend can be transformed to be increasing with the initial value to be zero.

The parameters of the mfWm are estimated based on the maximum likelihood estimation. The logarithmic maximum likelihood function is written as:

(26) $$\ln L(x|\lambda ,k)=n\ln(\frac{k}{\lambda })+n(k-1)\ln(\frac{1}{\lambda })+(k-1)\sum_{i=1}^{n}\ln x_{i}-{\left(\frac{1}{\lambda }\right)}^{k}\sum_{i=1}^{n}x_{i}^{k}.$$

The estimates for the shape parameter and scaling parameter can be obtained by solving Equations (27) and (28) numerically.

(27) $$\frac{\partial \ln L(x|\lambda ,k)}{\partial \lambda }=(\frac{-n}{\lambda })-n(k-1)(\frac{1}{\lambda })+k\sum_{i=1}^{n}x_{i}^{k}{\left(\frac{1}{\lambda }\right)}^{k+1}=0,$$

(28) $$\frac{\partial \ln L(x|\lambda ,k)}{\partial k}=n(\frac{1}{k})+n\ln(\frac{1}{\lambda })+\sum_{i=1}^{n}\ln x_{i}-\left[{\left(\frac{1}{\lambda }\right)}^{k}\sum_{i=1}^{n}x_{i}^{k}\ln x_{i}\right]-[\sum_{i=1}^{n}x_{i}^{k}{\left(\frac{1}{\lambda }\right)}^{k}\ln(\frac{1}{\lambda })]=0.$$

The parameter a connects the degradation states with the degradation speed. Defining the variable $X_{\Delta }(t)$:

(29) $$X_{\Delta }(t)=\frac{\dot{X}(t)}{X(t)},$$

the mean and variance of a are estimated from $X_{\Delta }(t)$.

The parameters b and c are estimated based on the fitting of the power function for the degradation trend. 

The drift coefficient controls the dispersion of the degradation rate. Therefore, the variance for the updating random walk is estimated from the historical degradation rate. The diffusion coefficient controls the changing rate of the degradation speed. The parameter κ connects the degradation speed and the changing rate. Defining another variable $X_{\kappa }(t)$:

(30) $$X_{\kappa }(t)=\frac{\ddot{X}(t)}{\dot{X}(t)},$$

the mean and variance of κ are estimated from $X_{\kappa }(t)$. 

## 4. Adaptive RUL Prediction Model on the Basis of the Degradation Model

### 4.1. Adaptive RUL Prediction Algorithm Based on the mfWm

The RUL is defined with respect to the first arrival time of the HI to the predetermined threshold. Denoting HI as X(t) to represent the degradation, the mathematical expression of the RUL can be formulated as follows:

(31) L(t)=inf{t:X(t+1)≥ω|X(t)<ω},

where w is the positive failure threshold (FT).

**Figure 5:**
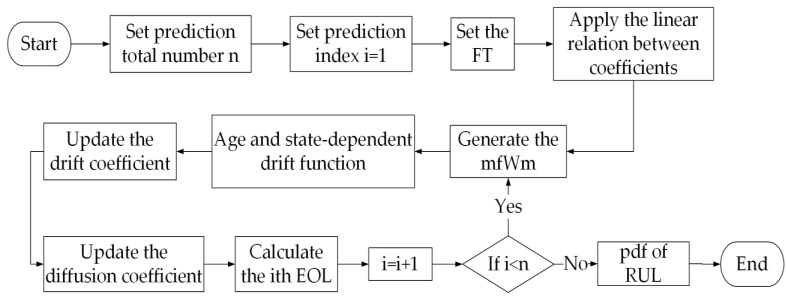
Flow chart of the adaptive RUL prediction algorithm.

The flow chart of the age and state-dependent degradation model with adaptive mechanism is depicted in Figure 5. The linear relationship between the drift coefficient and the diffusion coefficient is applied in the age and state-dependent degradation model. In the iteration, the mfWm is utilized to model the stochasticity of the degradation, and both the coefficients are continuously updated. When the degradation of HI first exceeds the FT, the lithium battery is considered to have reached its EOL. The degradation of HI contains stochasticity and uncertainty. Therefore, the point prediction is not the objective of the RUL prediction. Given a large amount of EOL predictions, the pdf of the RUL can be calculated by the Monte Carlo algorithm and the RUL with highest probability can be taken as the point prediction.

### 4.2. Convergence of the RUL Prediction Model

**Theorem** **2.** *The RUL prediction model based on mfWm is convergent.*

**Proof of Theorem** **2.** The values of the updated Gaussian noise are equally scattered around the origin. Therefore, the sum of the Gaussian white noise equals zero in the infinite time range.

The limitation of the drift coefficient is:

(32) $$\begin{array}{l} \underset{t\to \infty }{\lim}\mu (t)\\ =\underset{t\to \infty }{\lim}(1+\sum_{i=1}^{i=t}\varepsilon (t)),\\ =1, \end{array}$$

where $\varepsilon (t)∼N(0,\sigma_{\mu })$.

The degradation model is:

(33) $$X(t)=0.5\mu (t)\int_{0}^{t-1}a(s)X(s)ds+0.5\mu (t)b{\left(t-1\right)}^{c}+\kappa (t)\mu (t)mfWm(t).$$

The limitation for the degradation model is:

(34) $$\begin{array}{cc} \underset{t\to \infty }{\lim}X(t) & =\underset{t\to \infty }{\lim}0.5\mu (t)\int_{0}^{t-1}a(s)X(s)ds+\underset{t\to \infty }{\lim}0.5\mu (t)b{\left(t-1\right)}^{c}+\underset{t\to \infty }{\lim}\kappa (t)\mu (t)mfWm(t)\\ & =\underset{t\to \infty }{\lim}0.5\mu (t)\int_{0}^{t-1}\dot{X}(s)ds+\underset{t\to \infty }{\lim}0.5\mu (t)b{\left(t-1\right)}^{c}+\underset{t\to \infty }{\lim}\kappa (t)\mu (t)mfWm(t)\\ & =\underset{t\to \infty }{\lim}0.5X(t-1)+\underset{t\to \infty }{\lim}0.5b{\left(t-1\right)}^{c}+\underset{t\to \infty }{\lim}\kappa (t)mfWm(t). \end{array}$$

Assuming $\underset{t\to \infty }{\lim}X(t)=\underset{t\to \infty }{\lim}X(t-1)=S:$

(35) $$\begin{array}{cc} S & =\underset{t\to \infty }{\lim}b{\left(t-1\right)}^{c}+2*\kappa (\infty )mfWm(\infty )\\ & =\infty . \end{array}$$

Given a positive threshold ω, Equation (36) will converge to a positive integer:

(36) L(t)=inf{t:X(t+1)≥ω|X(t)<ω}.

Thus, we have proven that the RUL prediction model is convergent. □

## 5. Case Study

### 5.1. Lifetime Characteristics of the Lithium Battery in an Electric Vehicle

The lithium battery dataset here is a joint work of the Massachusetts Institute of Technology, Stanford and Toyota research center and has been updated on 16 June 2021 [50]. The data come from commercial lithium batteries for electric vehicles cycled under fast-charging conditions. These batteries have a nominal capacity of 1.1 Ah. The HI is the capacity, and the EOL is the cycle in which the capacity degrades to under 80% of the nominal capacity. All the batteries are tested in a 48-channel Arbin laboratory battery testing cycler, which is positioned in a forced convection temperature chamber with a steady temperature of 30 °C. 

**Figure 6:**
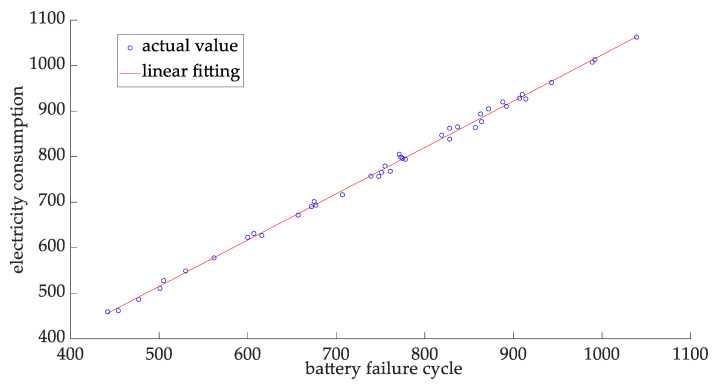
Linear relationship between lifetime and electricity consumption.

We utilize all the available lithium battery data in the Arbin cycler to demonstrate the lifetime characteristics. The lifetime values and electricity consumptions of the lithium batteries are plotted in Figure 6, which shows a clear linear relationship. Therefore, we can estimate the total electricity consumption of the electric car based on the RUL prediction, which provides a qualitative method to measure the impact of charging on the power grid. The expected mileage before the battery failure can be approximated based on the electricity consumption. The driver will be informed with the milage estimation for the replacement of the lithium battery before the fatal electric failure.

### 5.2. Lithium Battery Capacity Dataset for the Validation

**Figure 7:**
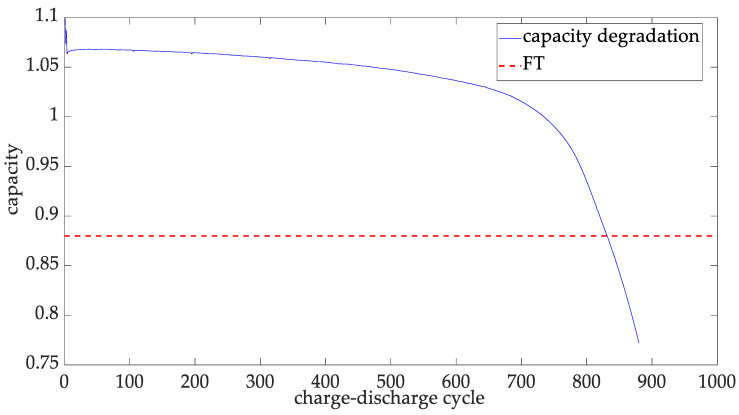
Capacity degradation of the lithium battery in channel 36.

In the case study, the lithium battery of channel 36 is used. The augmented Dickey–Fuller test is performed, and the results show the training set is stationary. As we can see from Figure 7, the battery capacity drops significantly in the first several cycles. After a long period of slow degradation, the capacity drops at high speeds because of the accumulated thermal and mechanical stresses. The red dotted line is the predefined FT. When the capacity of the lithium battery crosses the threshold, the battery is prone to fail and, thus, requires replacement for the safety of people and the reliability of the power system.

Gamma distribution, exponential distribution, log-normal distribution and the Weibull distribution are the common options for the modeling of HI. Here, we select two criteria to evaluate the fitting results: determination coefficient $R^{2}$ and chi-square coefficient $\chi^{2}$: a higher value of $R^{2}$ and a lower value of $\chi^{2}$ indicate better fitting results [51]. The formulas to compute them are given in Equations (37) and (38) below:

(37) $$R^{2}=1-\frac{\sum_{i=1}^{N}{\left(c_{i}-f_{i}\right)}^{2}}{\sum_{i=1}^{N}{\left(c_{i}-\bar{c}\right)}^{2}},$$

(38) $$\chi^{2}=\sum_{i=1}^{n}\frac{{\left(c_{i}-f_{i}\right)}^{2}}{f_{i}},$$

where $c_{i}$ and $f_{i}$ are the *i*th value of the capacity density function and of the fitted function. $\bar{c}$ is the mean value of the capacity density function.

The results of $R^{2}$ and $\chi^{2}$ for the statistical fittings are summarized in Table 1. As we see in Table 1, the fitting performance for the Weibull distribution is the best among the four distributions considered. Therefore, we chose the Weibull distribution as the fitting distribution.

### 5.3. Fluctuation of the Hurst Exponent in the Capacity Degradation Data

**Figure 8:**
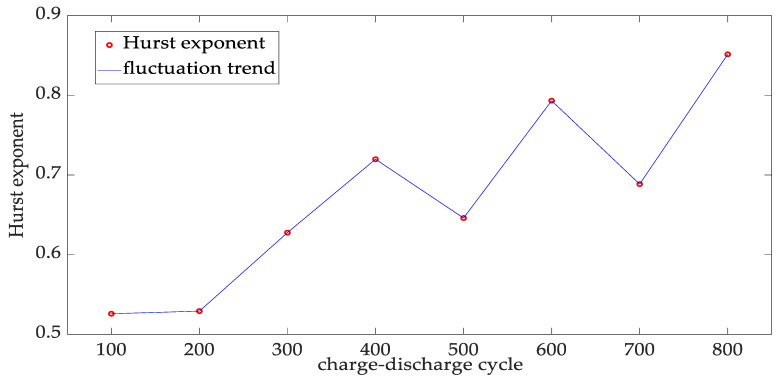
Fluctuation of the Hurst exponent in the capacity degradation data.

The Hurst exponent is used to characterize the long-range dependence of the stochastic series. The degradation data are stationary; thus, we choose the rescaled range algorithm [52]. We calculate the Hurst exponents every 100 cycles and plot the fluctuation in Figure 8. As we see in Figure 8, the Hurst exponents of the time series are fluctuating in the range of (0.5, 1), due to different operation conditions. Therefore, the capacity data possesses varying degrees of long-range dependence.

In this paper, we use a uniform variable $H_{v}$ instead of a constant H to define the mfWm. The degree of long-range dependence in the degradation model changes during the iteration, which enables the model to be adaptive to the varying operation conditions in the lithium battery degradation.

### 5.4. 1/f Characteristics in the Frequency Domain of the Lithium Battery

**Figure 9:**
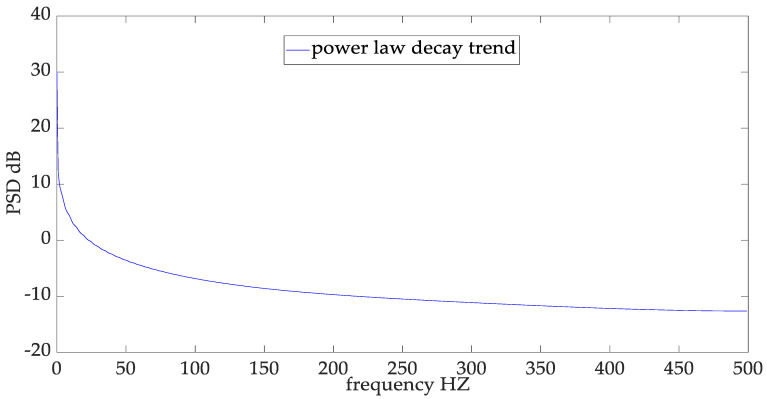
Power law decay for the PSD of the lithium battery.

The PSD of the lithium battery is plotted in Figure 9. The decaying rate of the PSD corresponds to a power law, which indicates that the capacity degradation is a 1/f noise in the frequency domain.

The definition of the 1/f noise leads to Equations (39) and (40) below:

(39) $$PSD=k\frac{1}{f^{\alpha }},$$

(40) $$\begin{array}{cc} 10\mathrm{lg}PSD & =10\mathrm{lg}(k\frac{1}{f^{\alpha }})\\ & =10\mathrm{lg}k-\alpha 10\mathrm{lg}f \end{array},$$

where α∈(0,2).

**Figure 10:**
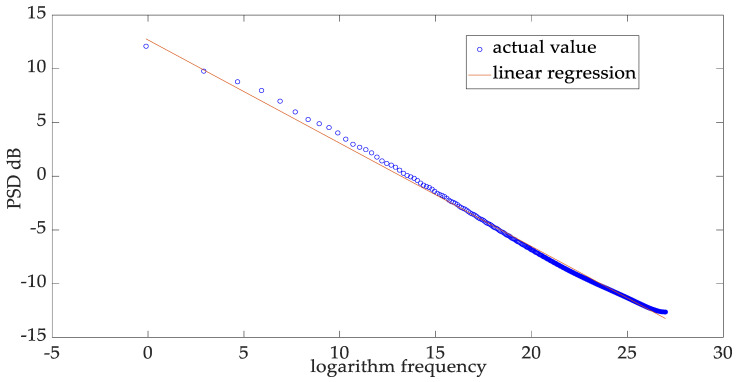
Logarithmic linear regression of the PSD.

The value of α can be calculated through logarithmic linear regression. In Figure 10, the parameter α is calculated to be 0.9607, which coincides with the 1/f characteristics of the mfWm. This means that the mfWm is suitable to model the degradation process of the battery capacity dataset.

### 5.5. Performance Evaluation of the mfWm Predictive Model

**Figure 11:**
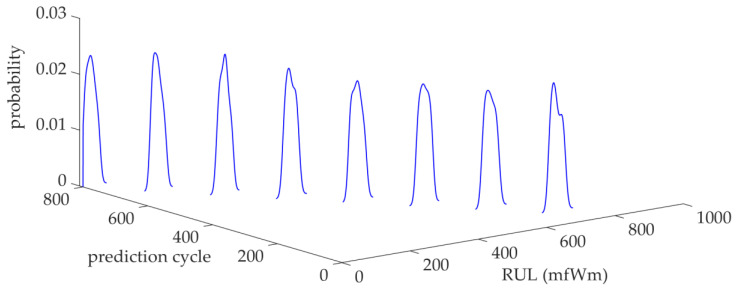
RUL prediction results of channel 36 based on the mfWm.

**Figure 12:**
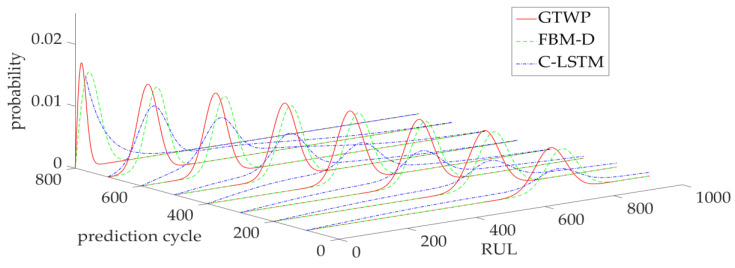
RUL prediction results of channel 36 for comparison.

The RUL prediction based on mfWm is depicted in Figure 11. For comparison, the prediction results of other algorithms are summarized in Figure 12. The point prediction is the mode of the RUL prediction results. In Table 2, the maximum, mean and standard deviation (std) of the relative error are provided, as well as the mean absolute error (MAE). The prediction errors for the GTWP and FBM-D are higher because they cannot express the varying degree of long-range dependence of the battery degradation. As we can see, the dispersion of the pdf is much larger for the C-LSTM method, which is mainly caused by the insufficiency of the training data. 

## 6. Conclusions

In this work, the mfWm is employed for the adaptive RUL prediction model of electric vehicle lithium batteries. The mfWm is suitable to describe the varying degree of long-range dependence of the battery degradation, which is caused by the change of operation conditions. The 1/f characteristics of the mfWm coincide with the lithium batteries in the frequency domain. As the mode of degradation shifts, the degradation rate and variational speed are continuously changing. Thus, the age and state-dependent degradation model based on mfWm updates the drift and diffusion coefficients on each iteration step. On the basis of the degradation model, the adaptive RUL prediction model is proposed for the electric vehicle lithium batteries with convergence.

The drift function in this work is age and state-dependent, which is more general than the time-dependent drift function. However, the drift function can be improved. Our future work will apply more advanced drift functions to the RUL prediction of the rotary machinery. 

## Figures and Tables

**Table 1:** Deterministic coefficients and chi-square values of the statistical fitting.

	Weibull	Gamma	Exponential	Log-Normal
$R^{2}$	0.6102	0.2375	0	0.25
$\chi^{2}$	993	1283	10351	1811

**Table 2:** Statistical metrics of the predictive error.

	Maximum	Mean	Std	MAE
**mfWm**	0.0349	0.0246	0.0107	20.5
**GTWP**	0.0541	0.0310	0.0249	30.4
**FBM-D**	0.0509	0.0292	0.0238	27.6
**C-LSTM**	0.0434	0.0250	0.0176	22.8

## Data Availability

The lithium battery data used in the research is available in https://data.matr.io/1/ (accessed on 1 March 2023).

## Abbreviations

RUL	remaining useful life
pdf	probability density function
HI	health indicator
EOL	end of life
C-LSTM	cocktail long short-term memory network
GTWP	general time-varying Wiener process
FBM-D	fractional Brownian motion with dynamic properties
mfWm	multi-fractal Weibull motion
PSD	power spectrum density
FT	failure threshold
std	standard deviation
MAE	mean absolute error
